# Discovering and accounting for limitations in applications of theories of word reading acquisition

**DOI:** 10.3389/fpsyg.2014.00579

**Published:** 2014-06-13

**Authors:** G. Brian Thompson

**Affiliations:** School of Educational Psychology and Pedagogy, Victoria University of WellingtonWellington, New Zealand

**Keywords:** reading acquisition theory, beginner reading, reading instruction, learning to read, reading vocabulary, letter sounds, phonological recoding, orthographies

More attention to the discovery of the limitations of current theories of word reading acquisition would enable progress in development of theories with a wider and more varied range of valid and useful applications. This general opinion is illustrated here with work that makes such an attempt.

There have been recent occasional attempts to apply computational connectionist models of adult word reading in simulations of children's normal progress in word and pseudoword reading (Hutzler et al., 2004; Powell et al., 2006), but they failed unless modifications were made that included adding “context-free” letter-sound correspondences to the initial training of the model. Taught phonics sounds for letters are such, as they are not bound to features within a word, such as position and/or the context of other (adjacent or otherwise) letter-sound correspondences of the word. Adding the phonic sounds was justified as a representation of the way children learnt because it was how they were taught reading. This introduces a major potential limitation in application of the model, in so far as it was improved for only one type of teaching, that with phonics. A new multiple-route theory (Grainger et al., 2012) of learning to read words has been proposed which may appear to avoid that problem but much of the learning of the beginner reader is modeled as in the theory of Share (1995). This, however, also requires full knowledge of “context-free” letter sounds for the initial development of word reading (Share, p. 164), as does the widely recognized theory of Ehri (1999, 2005, 2012). The illustration for my opinion focuses on the discovery of the limitations of this feature common to these theories, and on development of theory that accounts for evidence beyond the limitations.

## Failure in application of letter-sound requirements for development

There has been a claim (Thompson and Johnston, 1993; Ramus, 2004) of potential limitations of testing theories of reading acquisition on mainly those children receiving teaching in just the tradition in which the theory was developed. We cite data of participants from a teaching tradition very different from that in which the Share and Ehri theories were developed. A tradition has been common across New Zealand (since the late 1960's) in which neither context-free letter sound knowledge or explicit phonics were taught, and the emphasis was on text-centered teaching (Thompson, 1993) with individualized provision of multiple brief story texts at finely adjusted difficulty levels. In that country a sample with normal word reading progress, and 9 months of reading instruction, obtained a mean accuracy of 83% for names of the lower-case alphabet letters, 76% accuracy for the context-free phonic letter sounds of the 9 letters (*b, d, j, k, o, p, t, v, z*) with a name having the initial pronunciation element compatible with that phonic letter sound, but 51% accuracy for the sounds of the other 16 letters without such compatibility with the letter name. (Calculated from Thompson et al., 1999, that specifies the range of pronunciations obtained, and those acceptable as letter sounds, among these children who were not taught them. The letter *q* was not included due to the high rate of visual confusion with *p*.) A sample of 11-year-olds with normal progress (relative to both local and U.S. norms) in the same school system and tradition of teaching obtained a mean accuracy of 99% for the letter names, 90% for the sounds of the 9 letters compatible with the letter name, but 62% for the sounds of the 16 not compatible (Fletcher-Flinn and Thompson, 2004, p. 315). Moreover, a sample of adult university students with above average reading skill (relative to U.S. norms), who as children had been taught in that tradition in this school system, showed a similar result (Thompson et al., 2009). The conclusion is that successful readers in this teaching tradition did not meet the requirement of the theories (cited above) for full knowledge of context-free letter sounds for success in acquiring word reading.

## What accounts for this failure in application?

Aside from letter-sound knowledge, it may be that knowledge of letter identities *per se* can be acquired within the context of words, as children begin learning to read. In a series of studies relating to letter identities, 5-year-old children, after 9 months of reading instruction with normal progress, could cope fully with the substitution of upper case for lower case in their knowledge of identities of letters out of the context of words. The children responded to upper-case letters comprising those eight that were visually dissimilar to the corresponding lower-case form (Aa, Bb, Dd, Ee Gg, Hh, Nn, Rr) with accuracy as high as the lower case and very close to ceiling (Thompson and Johnston, 2007). But, in reading identification of familiar print words comprising these upper-case letters, the children were much less accurate than their high accuracy for the same words in lower case. Moreover, these results were replicated across children in the New Zealand teaching tradition and that in Scotland with explicit phonics. Other evidence in the study showed that these children were using some form of letter identities to read the words, rather than global visual features of the words. In another study, an experiment with training initially unknown and similarly constructed lower case words had similar effects in which gains in lower case accuracy were large but transfer gains for upper case were much smaller. This was despite equal proficiency in knowledge of identities of the letters in the two case forms when out of the context of words (Thompson et al., 2008). Hence, the processes for letter identities that are bound to word context for identification of words can function differently from those for “context-free” letter identities (Thompson, 2009).

Such a difference in processes may also be expected to occur for the beginner reader's use of letter-sound knowledge. In a sample of normal-progress New Zealand 5- and 6-year-olds there was evidence they had some knowledge of letter-sound relations that was bound to a sublexical function of their emerging *reading vocabulary* (which is the stored knowledge of the letters of the word, i.e., the lexical orthographic representation, along with the associated phonological and lexical-semantic representations). The children's relative accuracy of letter-sound relations in their reading of simple pseudowords (e.g., *ob, bu, et*, … that simulate new print words) was predicted from the distribution of occurrence of within-word positions of these sublexical relations among the vocabularies of the children's reading books. For example, these small vocabularies rarely included words with a final *b* letter, although an initial *b* was common, whereas *t* in both final and initial positions was common. The children's pseudoword reading accuracy reflected these (and similar) distributions of the positional sublexical letter-sound relations in their print word experience. They gave no segmented pronunciation of component letters, contrary to what would be expected in an explicit phonics response. Moreover, a replication of the task was conducted, and also confirmation by a successful prediction of positive effects (relative to controls) on pseudoword reading accuracy from experimental training that introduced words with final *b* into the children's reading vocabularies (Thompson et al., 1996). For children receiving explicit phonics instruction there has not been a complete replication involving the training experiment. The pseudoword reading task, however, was presented to such a sample of children in the U.S. who were of a comparable reading level, with the result showing no significant within-word positional effects (Fletcher-Flinn et al., 2004). Context-free letter sounds have no coding of position or other contextual feature of words. Hence, this result was expected for their phonological decoding of the pseudowords, if they were often using taught context-free letter sounds, rather than knowledge of letter-sound relations bound to a sublexical function of their emerging reading vocabularies.

These results are consistent with the Knowledge Sources theory that was developed to include different sources of knowledge to account for the acquisition of word reading in a tradition of text-centered teaching as well as a tradition with explicit phonics (Thompson et al., 1996; Fletcher-Flinn and Thompson, 2004; Thompson and Fletcher-Flinn, 2006, 2012). In this theory, as soon as the child, with support from parent or teacher, has acquired reliable reading of a few words, and has attended to “the relationship in which letters of words often match sound units of the spoken word” (Thompson and Fletcher-Flinn, 2012, p. 254), they can independently extract from their emerging reading vocabulary some letter-sound information coded with sublexical features. This coding can commence for position within the word and then expand to include the contexts of other letter-sound correspondences within the word (Thompson et al., 1996; Thompson and Fletcher-Flinn, 2006). Such sublexical information is available for a frequently implicit form of “phonological recoding” (involving generation of responses to new print words) that does not require full knowledge of context-free letter sounds, as in the theories of Share or Ehri. This form of phonological recoding assists the child in acquiring representations of new or unfamiliar print words, thus extending the child's reading vocabulary, which in turn is a basis for extracting more advanced sublexical letter-sound knowledge. It implies a recursive process that can start very early in the child's development of reading. The theory, however, also accounts for children's successes in using, as in explicit phonics, the other form of phonological recoding that is initially dependent on full knowledge of context-free letter sounds.

There are other studies, which examine samples of beginner readers who have reached the same developmental level of word reading but have differences in processes of word reading acquisition according to whether they are receiving teaching with explicit phonics or text-centered teaching (Connelly et al., 2009). In a study involving three countries (Thompson et al., 2008) the teaching with explicit phonics produced beginner readers who had much higher accuracy in pseudoword reading than those receiving text-centered teaching, although both had reached the same level of word reading accuracy. Nevertheless, for reading text, the context in which most useful word reading occurs, teaching with explicit phonics produced a much slower speed of text reading (for equal word reading accuracy). This was apparently not due to their slower responses to the unfamiliar words but mainly to their lower level of practice with words in text, and hence with the lexical-semantic and syntactic relations among the words. It is consistent with the Knowledge Sources theory to infer that the corresponding greater number of exposures to print words from text, along with the associated orthographic-phonological, orthographic-lexical, and lexical-semantic/syntactic relations, reduces the need for developing a high level of expertise in a form of phonological recoding initially based on knowledge of context-free letter sounds.

The specific focus here has been on success and failure of several theories in accounting for differences in beginner learning processes arising from varied traditions for initial teaching of reading. Knowledge Sources theory, however, has been discovered to have valid applications beyond that. The theory has been applied to a form of alphabetic orthography very different from English. Within Japanese hiragana there is a secondary phonemic function for which there are 36 *yoo-on* symbols, which are formed from some of the basic symbols that otherwise represent syllables (Coulmas, 2003). Beyond the application limits of other theories, Knowledge Sources theory accounted for results from training experiments on the initial learning of both Japanese beginners and second-language learners, as well as evidence from skilled hiragana readers on the generalization limits of their implicit knowledge of the formation principle for this phoneme representation within hiragana (Fletcher-Flinn et al., 2014). The theory has also been applied to the case of a 3-year-old precocious reader. Beyond the limitations of the other theories, the recursive learning processes that use sublexical information from the child's emerging reading vocabulary accounted for this child's underdeveloped context-free letter sounds (Fletcher-Flinn and Thompson, 2000, 2004).

By discovery of one limitation of some current theories as applied to children in a teaching tradition outside that in which those theories were developed, an alternative theory was formed that offers a tested account of reading acquisition with a wider and more varied range of applications. This is just one illustration. Other limitations await discovery in these and other theories.

### Conflict of interest statement

The author declares that the research was conducted in the absence of any commercial or financial relationships that could be construed as a potential conflict of interest.
